# Derisking Phase II Clinical Trials in Heart Failure: A Position Paper from *JACC: Basic to Translational Science* and the Heart Failure Collaboratory

**DOI:** 10.1016/j.jacbts.2025.04.003

**Published:** 2025-05-26

**Authors:** Douglas L. Mann, G. Michael Felker, Ellis F. Unger, Janet T. Wittes, Biykem Bozkurt, Fady I. Malik, Norman Stockbridge, Benjamin R. Saville, Mona Fiuzat, Christopher M. O’Connor, Scott D. Solomon

**Affiliations:** a Washington University School of Medicine, St. Louis, Missouri, USA; b Duke University Medical Center, Durham, North Carolina, USA; c Hyman, Phelps and McNamara, P.C., Washington, DC, USA; d Florida Atlantic University, Boca Raton, Florida, USA; e Winters Center for Heart Failure, Cardiology, Baylor College of Medicine and Michael E. DeBakey VA Medical Center, Houston, Texas, USA; f Cytokinetics, Inc, San Francisco, California, USA; g Independent Consultant, Dickerson, Maryland, USA; h Adaptix Trials, LLC, Austin, Texas, USA; i Vanderbilt University Medical Center, Department of Biostatistics, Nashville, Tennessee, USA; j Inova Heart and Vascular Institute, Falls Church, Virginia, USA; k Cardiovascular Division, Brigham and Women's Hospital, Boston, Massachusetts, USA

**Keywords:** clinical trials, heart failure, phase II

## Abstract

Phase II clinical trials play an important role in drug development, providing key data that guide decision-making for promising therapeutic candidates. An important objective of phase II is to establish proof of concept by demonstrating that the drug produces its intended biological effects in the target population. Phase II trials also evaluate pharmacokinetics, pharmacodynamics, safety, and dose-response relationships. Ultimately, the goal is to generate the evidence needed to inform go/no-go decisions for further development. However, as we will discuss, phase II studies have inherent limitations and cannot fully predict phase III outcomes. In this *JACC: Basic to Translational Science* and *Heart Failure Collaboratory* position paper, we examine the value and constraints of phase II programs, using prior examples of heart failure trials, with the goal of providing insights that will help investigators and sponsors to derisk go/no-go decisions.

Phase II clinical trials play an important role in drug development by generating information that informs decision making for a promising drug candidate. The objectives of phase II development are context-dependent but can be broadly categorized into several different areas. A primary goal of phase II trials is to establish proof of concept (PoC), by demonstrating that the therapeutic agent exhibits evidence of its intended biological activity in the target population. Phase II also assesses pharmacokinetics, pharmacodynamics, safety, and dose-response relationships. Ultimately, the goal of phase II is to provide the requisite information to make go/no-go decisions regarding further development of a therapeutic candidate. The decision to halt a drug development program is often difficult, insofar as abandoning development of a therapeutic candidate runs counter to the inherent bias to continue investing in an endeavor that has already consumed considerable time, energy, and resources (ie, the sunk cost fallacy^1^). This bias is further reinforced by the uncertainty of never knowing whether the outcome of a halted development plan might have been favorable had development continued. As will be discussed, phase II development programs have intrinsic limitations and will never perfectly predict outcomes in phase III trials. In this *JACC: Basic to Translational Science* and *Heart Failure Collaboratory* collaboration, we developed the following position paper to discuss the utility and limitations of phase II development programs using prior examples of phase II heart failure trials, with the goal of providing insights that will help investigators and sponsors to derisk go/no-go decisions.

## Phase II Clinical Trials

Phase I studies generate initial safety and pharmacokinetic data, typically beginning with healthy subjects before expanding to target patient populations. Phase II studies, discussed in detail in this paper, further evaluate safety and efficacy and dosing. Phase III studies aim to provide the evidence needed to support regulatory labeling for a new drug or an additional indication of an approved drug.

The U.S. Code of Federal Regulations *(21CFR312.21)* states, *“*Phase 2 (II) includes the controlled clinical studies conducted to evaluate the effectiveness of the drug for a particular indication or indications in patients with the disease or condition under study and to determine the common short-term side effects and risks associated with the drug. Typically, these trials follow successful Phase I studies. Phase II studies are generally closely monitored and conducted in a relatively small number of patients, usually involving no more than several hundred subjects.”^2^ Phase II trials range from exploratory (ie, screening, dose-finding) studies designed to generate preliminary insights to more rigorous investigations designed to confirm findings and justify further development (ie, decision-making).

Phase II studies have traditionally been divided into 2 categories: phases IIa and IIb; however, variations exist with respect to how they are defined and applied in different contexts. Phase IIa trials are focused primarily on selecting the dosing regimen. These trials, which aim to assess the pharmacodynamics and pharmacokinetics of the drug candidate in a patient population with the targeted condition, are usually larger than phase I trials. The goal is to identify an effective dose with an acceptable side-effect profile. Phase IIa studies typically include a smaller number of participants (in cardiovascular studies, 50-100 patients) than phase IIb studies and have limited statistical power to detect definitive efficacy outcomes. For this reason, phase IIa trial designs typically use biomarkers or other surrogate endpoints to support preliminary efficacy and dose selection for subsequent trials. These trials are designed to provide preliminary evidence of the therapeutic effect of the dose of the drug identified in phase IIa in patients with the target condition. Phase IIb trials often involve a larger number of participants (in cardiovascular studies, 100-500 patients) than phase IIa trials. Trial designs for phase IIb typically employ subjective clinical endpoints (eg, symptom improvement, disease progression) or validated surrogate markers to assess efficacy. They may include randomized controlled designs to compare the new treatment against a placebo or a standard treatment. These trials also continue to monitor safety and tolerability. The omecamtiv mecarbil program is a recent example of a drug development plan that progressed from phase IIa to IIb studies before proceeding to phase III clinical trials. A phase IIa dose-finding study employed a placebo-controlled cross-over dose ranging design.^3^ This was followed by the COSMIC-HF (Chronic Oral Study of Myosin Activation to Increase Contractility in Heart Failure) trial, which was a randomized placebo-controlled phase IIb study that assessed the pharmacokinetics and effects of omecamtiv mecarbil on cardiac function and structure of stable, symptomatic chronic heart failure patients with reduced ejection fraction.^4^

Despite the conceptual appeal of conducting phase IIa and IIb studies sequentially, this approach may be inefficient in terms of time and resources. Hybrid phase IIa/IIb adaptive study designs integrate the objectives of both phase IIa and IIb trials by combining dose-finding and efficacy assessment within a single trial framework. These designs aim to streamline the drug development process, reduce the number of patients required, and shorten the duration of clinical trials. In a hybrid phase IIa/IIb adaptive design, the initial part of the trial (phase IIa) focuses on identifying a desirable dose by evaluating the pharmacokinetics, pharmacodynamics, and preliminary safety in patients with the condition to be treated. Based on interim analyses, the study adapts by selecting what appears to be optimal doses and expanding participant numbers to confirm efficacy (phase IIb). Thus, the phase IIb portion assesses the efficacy of the selected dose in a larger patient population while continuing to monitor safety. Although hybrid phase IIa/IIb adaptive study designs are employed frequently in oncology studies, they have not been extensively used in heart failure drug development programs. GenePHIT is a phase II adaptive, double-blind, placebo-controlled, randomized, multicenter trial that evaluates the efficacy, safety, and tolerability of AB-1002, a gene therapy candidate, in patients with nonischemic cardiomyopathy and NYHA functional class III heart failure symptoms.^5^ AB-1002 is a genetically engineered adeno-associated virus construct designed to deliver a constitutively active form of protein phosphatase inhibitor-1 (I-1c) to the heart, with the goal of restoring intracellular calcium homeostasis in failing cardiac myocytes. Participants are randomized in a 1:1:1 ratio to receive a single injection of: low-dose AB-1002, high-dose AB-1002, or placebo. Following treatment, the trial includes a 52-week observation and a 4-year follow-up period. The trial incorporates an interim analysis to identify the more effective and safer dose of AB-1002 for further study.

## Predictive Accuracy and Success Rates of Phase II Clinical Trials

A recent study conducted by the Biotechnology Innovation Organization, Pharma Intelligence Informa, and Quantitative Life Sciences Advisers revealed that success rates for phase I and III development were notably higher than for phase II.^6^ Development during phase II emerged as the primary limiting step in the translation from bench to bedside, with PoC established in only 29% of drug programs at this phase. Notably, cardiovascular drugs exhibited a high failure rate in phase II transitions for novel drug candidates, with a success rate of ∼21%.^6^ At the time of this writing, no information is available with respect to success rates for novel drug candidates in phase II heart failure trials.

Some of the problems with the predictive accuracy of phase II cardiovascular studies stem from intrinsic challenges to phase II studies in general (eg, small sample sizes, patient heterogeneity, and limited follow-up); however, some of the problems also relate to how clinically meaningful endpoints are structured and analyzed in phase II, which can lead to erroneous interpretations of the data. Another consideration regarding the assessment of predictive accuracy is that once a development program is suspended at phase II, it is never known whether the therapeutic might have been successful in phase III had protocol adjustments been made based on phase II findings.

## Defining the Goals of Phase II Clinical Trials

The most important issue to address before designing a phase II clinical trial is to determine what information needs to be gathered to plan phase III and gain reasonable confidence that the therapy will show benefit in phase III. The collection and analysis of such data provide the basis for a decision algorithm that includes proceeding to phase III, performing another (phase IIb) study to gather additional information, or terminating development. Such decisions will obviously depend, in part, on what is already known about the therapeutic agent from prior nonclinical and clinical studies. Accordingly, the metric of success for phase II clinical trials should be whether the results of phase II trials provide investigators and sponsors with the ability to make accurate and informed decisions about proceeding to phase III, and not necessarily by the more conventional (narrow) benchmark of the number of phase II development programs that proceed to phase III or whether phase II results predict regulatory approval after phase III. For the purpose of the present discussion, we assume that the Investigational New Drug application for the drug has successfully cleared the U.S. Food and Drug Administration (FDA) review process and that the phase I development has demonstrated safety and tolerability at the doses tested in healthy volunteers, with acceptable toxicity.

The subsequent discussion examines the specific types of information that should be collected during phase II clinical trials to inform go/no-go decisions regarding progression to phase III clinical trials, recognizing that the types of information needed will not be the same for all therapeutic agents.

### Establishing PoC

One of the primary goals of any phase II clinical program is to establish PoC that the drug demonstrates the desired biological or clinical activity in the target population. Phase II PoC studies are often fraught with challenges that can complicate the interpretation of results, potentially jeopardizing the success of subsequent phase III development. Although nonclinical models (eg, animal studies or in vitro experiments) provide initial experimental evidence of biologic effect, they rarely replicate the complexity of human physiology and disease and rarely account for the background pharmacologic milieu. This is especially true in the broad clinical syndrome of heart failure, where multiple etiologies and concomitant treatments contribute to substantial heterogeneity. Moreover, the results of nonclinical models often do not provide information about the types of clinical endpoints that would be most meaningful in clinical studies. This is particularly the case for nonclinical heart failure models, which typically employ acute cardiac injury, which do not faithfully mimic the biology in patients with chronic heart failure.

PoC studies depend on carefully selecting endpoints that accurately capture the intended drug effect. These endpoints must balance sensitivity and variability to ensure a feasible sample size. In many cases, surrogate endpoints (eg, biomarkers, cardiac hemodynamic measurements, imaging results) are used instead of clinical endpoints (eg, 6-minute walk distance, heart failure hospitalization) because sample sizes can be smaller and dose ranging is more feasible. The ability of surrogate endpoints to predict efficacy, however, is not always borne out in phase III trials. N-terminal pro–B-type natriuretic peptide (NT-proBNP), often used as a surrogate endpoint in phase II heart failure trials, warrants special mention because it has substantial variability; therefore, results from even modest-sized studies can be inconclusive or misleading. Both pathway-specific and independent biomarkers can be of value in drug development. The former can validate the mechanism of action of the drug, whereas the latter can provide a broader perspective on clinical outcomes and systemic effects. For example, measuring cyclic guanosine monophosphate levels after treatment with drugs that activate nitric oxide signaling pathways (eg, vericiguat) may also lead to lowering NT-proBNP levels as an independent biomarker associated with improved clinical outcomes.^7^

The identification of target-specific predictive biomarkers (eg, lowering high sensitivity C-reactive protein levels with interleukin-6 antagonism) is still an evolving science. Moreover, target-specific biomarkers are not always available when phase II trials begin. In addition, although a biomarker may predict successful outcomes in phase III trials for a specific drug, it may not reliably predict successful phase III outcomes for other drugs. For example, changes in NT-proBNP levels in the phase II PARAMOUNT (Prospective comparison of ARNI with ARB on Management Of heart failure with preserved ejection fraction) trial^8^ predicted modest success in the phase III PARAGON-HF study (Prospective Angiotensin Receptor Antagonist Global Outcomes in Heart Failure), insofar as PARAGON-HF fell just short of meeting its primary endpoint.^9^ In the PARADIGM trial, on the other hand, NT-proBNP reduction was an accurate surrogate for the outcomes benefit.^10^ In contrast, although empagliflozin led to no change in NT-proBNP levels in the EMPERIAL-Reduced (Empagliflozin outcome trial In patients with chronic heart failure with reduced ejection fraction)^11^ or EMPERIAL-Preserved trials (Empagliflozin outcome trial In patients with chronic heart failure with preserved ejection fraction),^12^ both the phase III EMPEROR-reduced^13^ and EMPEROR-preserved^14^ clinical trials showed significant improvements in the primary endpoint of cardiovascular death and heart failure hospitalization.

Identifying the appropriate patient population is critical to demonstrating PoC. Patient heterogeneity is a persistent challenge in PoC studies, including in heart failure. Selecting patients who are more likely to benefit from the therapy based on genetic or phenotypic factors (eg, baseline left ventricular ejection fraction or 6-minute walk distance) can increase the observed effect size. This approach, known as predictive enrichment, can help improve study outcomes by focusing on a more responsive patient population. In small phase II heart failure studies, patient heterogeneity and confounding comorbidities can contribute to variability that makes demonstration of PoC particularly challenging. Moreover, phase II trials are typically performed in fewer clinical sites with a more limited geographic distribution than typical outcome trials, and the results may be less generalizable to the more global population.

### Dose selection

Although demonstration of PoC is a central focus of phase II clinical trials, dose selection is also a critical objective. Phase IIa studies assess safety, tolerability, pharmacokinetics, and preliminary efficacy to identify the dose or doses that are most likely to be effective with an acceptable adverse event profile. By refining and narrowing the dosing options, phase IIa reduces the likelihood of testing suboptimal doses in later stages, which could jeopardize success. Phase IIb typically includes larger randomized, controlled studies that build on data obtained in phase IIa, generating more robust data on safety and clinical endpoints or surrogate markers, all of which aid in designing the phase III program. Dose selection may continue in phase IIb and, in some cases, in phase III.

### Balancing safety and efficacy

Although PoC studies typically focus on identifying efficacy signals, striking the right balance between safety and efficacy in phase II is essential to making informed decisions about advancing to phase III. Phase II trials are conducted in sample sizes large enough to assess responses in relatively diverse clinical populations with the target indication, and importantly to provide an initial characterization of safety in a disease population. Often, phase II studies provide the best opportunity to evaluate adverse events as a function of dose, providing data that can help assess whether adverse events are drug-related. In trials that enroll patients with advanced cardiovascular disease, many adverse events are likely to occur that are unrelated to the study drug, which can make interpretation of safety difficult. Assumptions regarding drug-relatedness of such adverse events can be incorrect and counterproductive; however, it is important to take all adverse events seriously and not dismiss their significance. Adverse events that occur infrequently typically require larger sample sizes for detection and characterization, which can be achieved in large phase III trials.

Safety monitoring is another important consideration for phase II studies. The FDA states, “All clinical trials require safety monitoring, but not all trials require monitoring by a formal committee that may be external to the trial organizers, sponsors, and investigators.”^15^ Although the FDA does not mandate the use of Data Monitoring Committees (DMCs) in phase II clinical trials, it recommends their use in certain situations to enhance participant safety and data integrity. Specifically, the FDA suggests considering a DMC for trials that are blinded, involve high-risk interventions, or include vulnerable populations. The decision to utilize a DMC should be based on factors such as trial complexity, potential risks, and the need for independent oversight. For detailed guidance, refer to the FDA document on the establishment and operation of clinical trial data monitoring committees.^15^

## Phase II Clinical Trial Design

**Figure 1:**
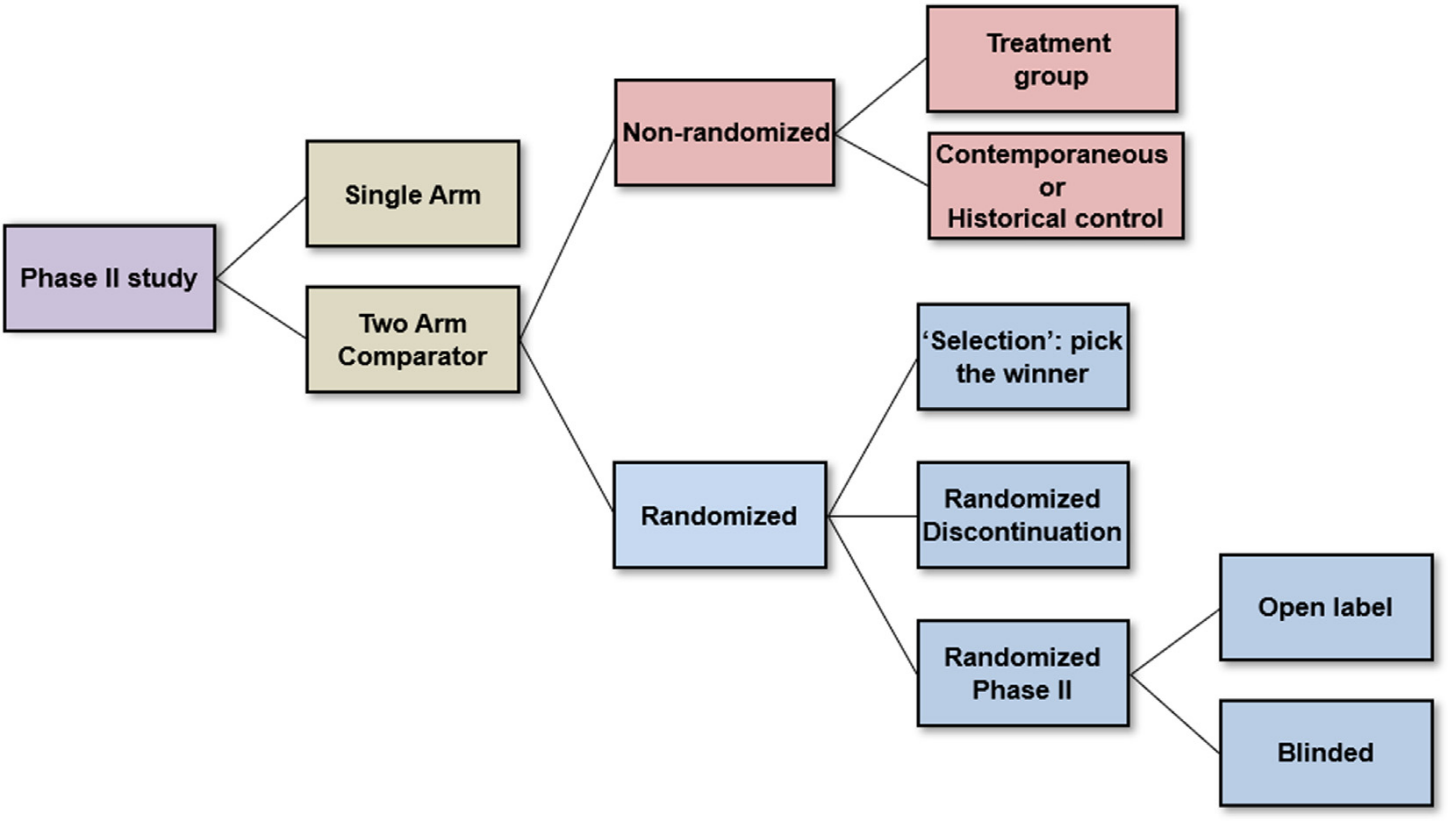
**Types of Phase II Trial Designs** Modified and reproduced with permission from Seymour L et al. Trial Design Task Force of the National Cancer Institute Investigational Drug Steering Committee. *Cancer Res.* 2010:16;1764-1769.

As shown in Figure 1, phase II clinical trials may be either single-arm or 2 or more arms. Single-arm studies are typically “externally controlled” (ie, lack a concurrent control group) and, although common in oncology trials, are unusual in trials of cardiovascular disease. Such trials rely on comparisons to response rates observed in external databases. They are typically reserved for rare diseases where few subjects are available for enrollment, or studies where invasive procedures are required for delivery of the therapeutic agent and use of a placebo control group would be unethical. Thus, an important question in the design of phase II trials is whether randomization in a two-arm comparator trial is feasible.

The control group may be a placebo or an active drug (active-control trial). Adaptive clinical trial designs may span phases IIa and IIb or phases IIb and III, allowing for seamless transitions between early- and late-stage evaluations of a therapy. Adaptive designs enable modifications to trial parameters (eg, dosing regimens, sample size, enrollment criteria) based on interim analyses, thereby increasing efficiency and decreasing resource utilization. By incorporating adaptive elements, these trials can accelerate drug development while maintaining rigorous scientific and regulatory standards. In certain situations where a safe, effective dose is known and safety is well characterized (eg, when an approved drug is being evaluated for a new indication), it may be appropriate to transition directly from phase I to clinical phase III trials. One recent example of this approach was the PARADIGM-HF trial,^16^ where the safety and dose of LCZ696 (sacubitril/valsartan) were already known from prior hypertension trials.^17^ Single-arm phase II trials comparing baseline and post-treatment clinical status are sometimes used for evaluating drugs in very rare diseases (eg, Danon disease), but are often inadequate for identifying compounds with the potential to succeed if phase III trials are required for approval. This limitation arises because of the complexity of heart failure pathophysiology and the substantial variability in therapeutic responses caused by patient heterogeneity, as well as the presence of multiple comorbidities.

## Statistical Plans for Phase II Clinical Studies

No one-size-fits-all statistical approach is appropriate for phase II heart failure trials, insofar as the choice of methodology will depend on the types of questions that are being asked and the nature of the endpoints. Frequentist statistical methods that focus on a single primary endpoint remain the most widely used approach and provide an established framework for hypothesis testing and confidence interval (CI) estimation. Newer frequentist and Bayesian composite approaches, such as the win-ratio,^18^^,^^19^ z-scores,^20^, ^21^, ^22^ and heat maps,^23^ are increasingly being used and allow multiple endpoints to be analyzed simultaneously. The composite approach provides a framework to consider the entire patient experience in evaluating treatment benefit, and derisks the chance of missing benefit on one of several key endpoints. Bayesian methods, which can incorporate prior knowledge into study design and analysis, have been utilized in phase III and pivotal heart failure development programs (eg, Cardiac Contractility Modulation^24^), but have not been widely used in phase II heart failure trials. However, there remains great opportunity to leverage Bayesian and adaptive trial methodologies in phase II heart failure trials to increase trial efficiency and derisk the go/no-go decisions into phase II. For example, Bayesian methods can be useful for dose-response modeling and adaptive arm dropping, sample size selection via prediction of long-term outcomes, study population enrichment based on responding subpopulations, modeling of surrogate outcomes to predict clinical outcomes, incorporation of external evidence via prior distributions (Bayesian borrowing), and seamless phase II/III trial designs. In rare disease settings in which randomization is challenging, Bayesian methods can be useful for comparing an intervention group vs external placebo data, or for comparing an intervention arm vs a randomized placebo arm that is augmented with external placebo data.

Often it is not clear how a therapy will impact traditional heart failure endpoints (eg, heart failure hospitalization, cardiovascular death). Accordingly, in phase II trials, it is often necessary to strike a compromise between the breadth of exploration and statistical certainty. Although discovery drives the identification of new therapeutic possibilities, reproducibility confirms that observed effects are consistent and reliable across diverse settings. In this context, the emphasis may shift away from achieving a statistically significant result on a formal hypothesis test (ie, *P* < 0.05), toward a more comprehensive assessment of therapeutic activity across multiple endpoints. This can be accomplished by assessing the totality of the data using z-scores or heat maps to standardize all of the available endpoints, subgroups, and exploratory analyses, to ascertain whether the findings present a coherent and compelling narrative.^20^, ^21^, ^22^ By calculating z-scores across all of the key endpoints (primary, secondary, and exploratory), investigators and sponsors can assess whether the effects are directionally consistent across multiple domains, or whether they vary significantly over multiple clinical domains. Heat maps visually summarize the magnitude and significance of effects across endpoints and subgroups and are helpful for visualizing patterns and inconsistencies in the data.^25^ Assessing the totality of the data may provide investigators and sponsors with important information that is not obvious with traditional frequentist statistical approaches.

Evaluating dose-response relationships for both efficacy and toxicity across more than 1 dose can provide valuable insights into the potential benefits and risks of a drug. The goal is to gather sufficient information to determine whether to proceed with a phase III program and, if so, to guide its design in terms of dose selection, endpoints, and the time required to observe meaningful drug effects. Importantly, even if a phase II trial does not achieve statistical significance on its primary endpoint, it can still yield important data that inform the feasibility and optimization of a larger confirmatory phase II trial. Phase II trials are unique in this regard, in that they provide an opportunity to define new and often previously unsuspected or unknown endpoints. Recognizing that there are inherent challenges of phase II trials, there is a need for data analysis plans that are both flexible and rigorous enough to support the discovery of meaningful endpoints that can be validated in subsequent studies.

## Safeguards to Help Derisk Phase II Clinical Studies

One important safeguard to help derisk phase II drug development programs is to develop a thoughtful study design and rigorous analysis plan. The use of randomization, double-blinding, and control groups helps to ensure that observed effects are unlikely to be caused by selection bias, chance, or confounding factors. A potential caveat to this statement is that in small phase II trials randomization may not result in treatment groups that are well-balanced with respect to baseline characteristics.^26^ As a result, unmeasured cofounders can potentially bias study outcomes. The risk of imbalances is greater in trials with unequal randomization, where a larger proportion of participants are assigned to the investigational treatment arm, which increases the likelihood of baseline imbalances. Thus, one inherent limitation of all phase II clinical trial designs is that trial sizes are often too small to ensure that randomization results in balanced groups. Stratified randomization or covariate-adjusted strategies can be employed to address these challenges but may not eliminate the occurrence of random baseline imbalances.

Phase II trials can provide preliminary data needed to calculate effect sizes and variability, which are crucial for performing power analyses for larger phase III trials. The law of large numbers is a fundamental theorem in probability and statistics that predicts as the sample size increases, the sample mean converges to the population mean. Thus, larger sample sizes will yield more reliable estimates of treatment effects. The sample size needed to ensure adequate balance also depends on the number and variability of factors that influence the outcome of interest. For example, in heart failure studies, beyond demographic factors, the etiology, baseline symptom severity, LV function, comorbidities (eg, diabetes, kidney disease), concomitant therapies, and even factors related to the geographic location of study sites may affect the outcome. Imbalances in these factors may contribute to variability in the effect size of the intervention. Although phase II studies indeed help inform the design of phase III trials, they rarely have sufficient numbers of outcome events to adequately estimate event rates for phase III. Thus, sample size calculations for phase III are often based on assumptions and external other data, even when phase II data are available. Alternatively, Bayesian approaches have been suggested to estimate effect sizes for the purpose of power calculation for phase III trials,^27^ although this approach has not yet been employed often in phase II heart failure trials. Such methods can formally account for the uncertainty in the phase II estimates, which may motivate the use of a flexible adaptive sample size in a subsequent phase III trial and derisk the chance of an underpowered confirmatory trial.

The statistical analysis plan should clearly specify the primary and secondary endpoints and include statistical criteria for assessing significance. A plan for controlling the type I error rate should be included if multiple endpoints are analyzed.^28^ The inclusion of appropriate sample size calculations helps reduce the risk of false positives or negatives. If a study is underpowered to detect a meaningful difference between 2 groups there is <25% chance that a positive finding is a true positive; accordingly, the finding would unlikely be confirmed in phase III.^29^^,^^30^ By powering the trial appropriately and prespecifying the endpoints and the statistical analysis plan, investigators and sponsors can increase the likelihood that the results will be substantiated in subsequent studies.

When results fail to show statistical significance of the primary endpoint, exploration of other endpoints can be informative; however, this approach has potential benefits and drawbacks that should be carefully considered to ensure scientific rigor and validity. Exploring secondary or exploratory endpoints can reveal meaningful trends or patterns that were not apparent in the primary analysis. These insights may generate hypotheses for future studies or suggest alternative mechanisms or effects. Analyses of dose-response and secondary or exploratory endpoints can provide a more nuanced understanding of the study outcome, offering a broader perspective on the potential risks and benefits of the intervention. Examining other endpoints may also reveal heterogeneity in responses across different subpopulations, which could inform personalized approaches to treatment. In some cases, where the primary endpoint did not reach statistical significance the analysis plan may identify a finding with a *P* value that is “nominally” statistically significant. Such results may arise from prespecified endpoints that included a plan to control the type-I error rate, or where alpha was reduced because of other prespecified statistical testing. Nominally significant *P* values may also arise from analyses of other endpoints where control of the type-I error rate was not planned. Such endpoints may have been prespecified as secondary, tertiary, or exploratory endpoints, or not prospectively planned but analyzed post hoc. Regardless of the positioning of the endpoint, such results should be considered exploratory, and the results should be interpreted with caution.

Ultimately, the best way to validate and derisk the findings in exploratory analyses of phase II trials is to conduct a subsequent phase II study to see if the findings can be confirmed. In the context of phase II development plans, this can be accomplished by performing sequential phase IIa and IIb studies. A second study allows investigators and sponsors to verify that the observed effects are less likely the result of chance, bias, or study-specific anomalies, thereby bolstering the evidence for causality. This approach, however, may raise the costs of the phase II program and potentially delay further development, which could affect the patent lifespan of the drug. Moreover, although conducting 2 phase II studies offers a rigorous and robust approach to confirming and derisking findings from exploratory analyses in phase II, even confirmation of a phase II study does not guarantee success in phase III trials, which typically enroll patients across multiple sites and geographic regions and include larger, more diverse populations. These factors can introduce variability in responses that cannot be predicted reliably from the phase II results.

The drug development plan for serelaxin provides an example of a successful phase II study that did not translate into a meaningful clinical benefit in a large phase III trial. The Pre-RELAX-AHF (Relaxin in Acute Heart Failure) study was a phase IIb dose-finding trial designed to evaluate the efficacy and safety of serelaxin (a recombinant human relaxin-2 [vasoactive peptide]) in patients with acute heart failure. Patients were randomized to receive a 48-hour intravenous infusions of 1 of 4 doses of serelaxin or placebo, in addition to standard care. Pre-RELAX-AHF did not have a prespecified primary endpoint but rather assessed the overall effect of intravenous serelaxin across several clinical domains. The study found that, when compared with placebo, a dose of 30 μg/kg/d of serelaxin improved dyspnea as assessed by a Likert scale and a dyspnea visual analog scale through day 14. In addition, treatment with serelaxin was associated with a reduction in cardiovascular death or readmission caused by heart or renal failure through day 60.^31^ Based on the promising results of this phase IIb trial, the phase III RELAX-AHF trial was conducted in 1,161 patients.^32^ The RELAX-AHF trial showed that treatment with serelaxin resulted in improvements in relief of dyspnea assessed by the area under the curve for the visual analog score through day 5 but not by the Likert scale during the first 24 hours. There were no statistically significant effects on the secondary endpoints of cardiovascular death or readmission to the hospital for heart or renal failure, or days alive and out of the hospital through day 60. However, treatment with serelaxin was associated with a significant reduction in death at day 180. These encouraging results led to the much larger (n = 6,800) phase III RELAX-AHF-2 trial, which sought to confirm these benefits.^32^ The primary endpoints for RELAX-AHF-2 were 180-day cardiovascular death and worsening heart failure through day 5. Despite the promising earlier results in the Pre-RELAX-AHF and RELAX-AHF trials, RELAX-AHF-2 failed to meet its primary endpoints. In addition, serelaxin did not significantly impact other secondary endpoints, including all-cause mortality, rates of rehospitalization, or length of hospital stay. The serelaxin drug development plan serves as a reminder that early-phase success does not always predict later-stage efficacy.

## Safeguards to prevent overinterpreting phase II results

Phase II clinical development programs rarely yield unequivocal results. Failure to attain statistical significance for the prespecified primary endpoints, borderline statistical significance for secondary or unexpected exploratory endpoints, variability in patient responses in different subgroups, or conflicting data between biomarkers and subjective clinical endpoints can make it difficult to provide clear go/no-go signals with respect to proceeding to phase III. In such cases, investigators and sponsors face difficult decisions about whether to proceed directly to phase III, modify the drug or trial design, conduct a second phase II trial, or terminate the program entirely. Recognizing that no single approach guarantees a clear translational path forward to successful outcomes in phase III, we provide several ideas to help interpret ambiguous results of phase II clinical trial results.

The most important safeguard to prevent overinterpretation of equivocal phase II results is intellectual honesty, especially if there is uncertainty about the safety of an intervention. Investigators, sponsors, and journal editors must all resist the temptation to overinterpret or selectively emphasize data that align with desired outcomes. Instead, they should acknowledge ambiguity where it exists and evaluate both positive and negative trends in the data without bias. Intellectual honesty also requires recognizing the uncertainty in the data and limitations of the study, such as small sample size, variability, inadequate blinding, or methodological issues, which may contribute to equivocal findings. Such transparency will help to ensure that follow-up studies are informed by a realistic understanding of the data, rather than overly optimistic interpretations that will unravel and become apparent when tested in larger phase III clinical trials.

It is important to consider whether observations in endpoints that were not prospectively planned align with the known mechanism of action of the investigational agent. If the mechanism is unclear or contradictory, additional nonclinical studies may be warranted before engaging in further clinical studies. Moreover, in some cases, nonclinical translational studies may have significant limitations, leading to unreliable results.^33^ The cardiac stem cell trials in heart failure serve as a cautionary tale of how ignoring this fundamental principle in translational research led to inconsistent results in clinical trials. Cardiac stem cell therapies were initially proposed to regenerate damaged myocardium, restore contractile function, and improve outcomes in heart failure patients. When early clinical trials did not support this hypothesis, the hypothesis shifted to the paracrine effects of stem cells and finally to the release of extracellular vesicles (eg, exosomes) by stem cells. Despite the lack of clarity around the mechanism of action, the stem cell field advanced rapidly to large-scale clinical trials using different cell types, dosing strategies, delivery methods, and patient populations, all of which led to inconsistent clinical outcomes that stifled further development in the field.^34^^,^^35^

Another consideration is to examine the effect size of any observed drug effects closely and to assess whether the observed magnitude is clinically meaningful, and not just statistically significant. Small effect sizes in phase II trials may become even smaller in larger phase III trials when tested in broader patient populations.^36^ Moreover, if the magnitude of clinical benefit is small, it may not lead regulatory authorities to conclude that the benefit-risk profile is positive or satisfy the Centers for Medicare and Medicaid Services criteria for coverage, particularly when the therapy is expensive or its real-world impact is uncertain. Conversely, if the observed effect size is very large, it may indicate a groundbreaking new discovery, or it could instead suggest methodological issues, biases, or statistical anomalies. Understanding when an effect size is "too good to be true" and what constitutes a meaningful effect size is critical for evaluating the credibility and clinical relevance of findings. It is essential to recognize that the clinical significance of effect size is context-dependent; it varies according to the specific endpoints being assessed. A positive outcome in a clinical trial with a small sample size may be mistakenly interpreted as strong evidence of efficacy for a new therapy. For example, the surprising and nominally significant mortality benefit observed in the ELITE (Evaluation of Losartan in the Elderly Study),^37^ in which losartan showed a greater benefit than captopril in chronic heart failure, and in the RELAX-AHF trial,^32^ in which serelaxin appeared to show a mortality benefit in acute heart failure, were widely interpreted as evidence of benefit. In both instances, properly powered trials failed to show any evidence of mortality benefit.^38^^,^^39^ On the other hand, better than expected positive results in smaller sample size/relatively underpowered trials can also accurately predict outcomes in subsequent larger trials, such as the mortality reduction by carvedilol in the MOCHA (Multicenter Oral Carvedilol Heart Failure Assessment) trial^40^ foreshadowing the outcome in COPERNICUS (Carvedilol Prospective Randomized Cumulative Survival).^41^

Subgroup analyses in phase II trials may help to identify subpopulations that respond differently, whether defined by demographic factors, disease characteristics, genetic variations, comorbidities, or concomitant medications, which can inform the study design in phase III. Subgroup analyses may also help to identify safety issues (eg, elderly population, patients with renal impairment). The interpretation of subgroup results requires caution, however, especially in phase II studies where the subgroups are small. It is important to prespecify subgroups of great interest, and to explore only those subgroups that are large enough to provide reasonably reliable information. All relevant subgroups should be analyzed and reported to assess whether the effects in a promising subgroup are also trending in the same direction as the other subgroups. Consistency across subgroups strengthens confidence in the generalizability of the results and reduces the risk of chance findings or bias. For subgroups based on continuous variables (eg, age, body mass index), it is advantageous to categorize the subjects in tertiles or quartiles rather than dichotomize subjects at an arbitrary cut point, because the observation of a consistent trend across quartiles can lend confidence to the findings. Sometimes it is useful to analyze the outcome not within subgroups of a variable but rather as a function of the variable measured continuously. Conversely, discordant results among subgroups may signal that positive results in a randomly chosen subgroup are a chance finding. Discordant results may suggest the need for further investigation to understand the underlying biological or methodological factors that contribute to the discrepancies. In general, sample sizes in phase II trials are usually too small to provide confidence in subgroup results, although such results can be used for hypothesis testing in future trials.

In some cases, even when a phase II trial fails to demonstrate a statistically significant efficacy signal, investigators or sponsors may choose to proceed to a phase III trial based on observed trends in the data. This approach, while sometimes justifiable, carries significant risks. For example, SOCRATES-REDUCED (Study of the Effects of Vericiguat in Patients with Heart Failure and Reduced Ejection Fraction) was a phase IIb trial that evaluated vericiguat in patients with heart failure with reduced ejection fraction.^42^ The study did not find a statistically significant difference in the primary endpoint, which was a change in NT-proBNP levels between the vericiguat and placebo groups; however, an exploratory secondary analysis suggested a marginal dose-response relationship, with higher doses of vericiguat associated with greater reductions in NT-proBNP. Despite the lack of a robust finding in phase II, the sponsor decided to conduct the phase III VICTORIA (Vericiguat Global Study in Subjects with Heart Failure with Reduced Ejection Fraction) trial,^7^ which showed nominal statistical significance in reducing the composite endpoint of cardiovascular death and heart failure hospitalization. The effect size was, however, modest, which raised questions about the clinical importance of the findings. This example underscores the potential challenges and uncertainties in advancing therapies with marginal phase II results, emphasizing the need for careful consideration of the benefit-risk profile and the robustness of the data before committing to larger, costlier phase III trials.

## Summary

A successful phase II clinical development program should provide investigators and sponsors with enough safety and efficacy data to proceed to phase III clinical trials or to reach the decision to discontinue development without undue fear that stopping is premature. By demonstrating proof of concept, identifying safe and effective dose/dosing regimens, confirming satisfactory safety profiles, and refining patient selection criteria, phase II trials provide the framework for designing a successful phase III trial. The more robustly these goals are achieved in phase II, the lower the risk of failure in phase III, thereby saving time and resources and ultimately ensuring that effective therapies reach heart failure patients as efficiently as possible. As noted, however, for some clinical programs where the safety and dose/dosing of the drug are already well characterized, phase II clinical trials may not be necessary before proceeding to a well-powered phase III trial. Finally, no matter how well-designed a phase II development plan may be, it cannot guarantee success in phase III because of the inherent limitations of phase II study designs. These trials often rely on relatively small sample sizes, surrogate endpoints, or limited follow-up durations, which may not fully capture the complexities of the treatment effect in larger, more diverse patient populations. Thus, even the most thoughtfully designed phase II program carries an element of risk. However, as discussed in this position paper, there are a number of ways that investigators and sponsors can derisk phase II clinical trials.

## Funding Support and Author Disclosures

Dr Mann has received consulting fees from Cardurion, HAYA Therapeutics, Novo Nordisk Holdings, Tenaya Therapeutics, and Tourmaline Therapeutics; and has served on the Data Safety Monitoring Board for Impulse Dynamics. Dr Felker has received research grants from National Institutes of Health, Bayer, Bristol Myers Squibb, Novartis, Daxor, Merck, Cytokinetics, and CSL-Behring; has acted as a consultant to Novartis, Bristol Myers Squibb, Cytokinetics, Innolife, Boehringer Ingelheim, Abbott, Sanofi, Regeneron, Myovant, Sequana, Windtree Therapeutics, and Whiteswell; and has served on clinical endpoint committees or data safety monitoring boards for Merck, Medtronic, EBR Systems, Rocket Pharma, V-Wave, and LivaNova. Dr Unger is an employee of Hyman, Phelps and McNamara, P.C. Dr Wittes has received consulting fees from Bristol Myers Squibb, Celecor, Dalcor, Estar, GlaxoSmithKline, Keros, Kyverna, Madrigal, Novartis, Ophirex, Pulmocide, and Vertex. Dr Bozkurt has received consulting fees from Amgen, AstraZeneca, Bristol Myers Squibb, scPharmaceuticals, Baxter, Sanofi, Relypsa, Vifor, Roche, and Boehringer Ingelheim; has served on the steering committees of Relypsa and Renovacor; has served on a Clinical Event Committee for Abbott Pharmaceuticals; and has served on the Data Safety Monitoring Board for LivaNova Pharmaceuticals. Dr Malik is an employee of Cytokinetics. Dr Stockbridge has consulted for Attralus, BridgeBio, Edgewise Therapeutics, Eloxx Pharma, Gossamer Bio, Implicit Bioscience, Lexicon Pharmaceutics, Otsuka, Pahr, Renibus Therapeutics, Theravance Biopharma, United Therapeutics, Vascular Therapies, and Xylocor Therapeutics. Dr Saville is the owner of Adaptix Trials LLC, a statistical consulting company specializing in Bayesian and adaptive clinical trial design. Dr O’Connor has received consulting fees from Merck, Abiomed, and Zealcare. Dr Solomon has received research grants from Actelion, Alnylam, Amgen, AstraZeneca, Bellerophon, Bayer, Bristol Myers Squibb, Celladon, Cytokinetics, Eidos, Gilead, GlaxoSmithKline, Ionis, Lilly, Mesoblast, MyoKardia, National Institutes of Health/National Heart, Lung, and Blood Institute, NeuroTronik, Novartis, Novo Nordisk, Respicardia, Sanofi Pasteur, Theracos, and US2.AI; and has served as a consultant for Abbott, Action, Akros, Alnylam, Amgen, Arena, AstraZeneca, Bayer, Boehringer Ingelheim, Bristol Myers Squibb, Cardior, Cardurion, Corvia, Cytokinetics, Daiichi Sankyo, GlaxoSmithKline, Lilly, Merck, MyoKardia, Novartis, Roche, Theracos, Quantum Genomics, Cardurion, Janssen, Cardiac Dimensions, Tenaya, Sanofi Pasteur, DiNAQOR, Tremeau, CellProThera, Moderna, American Regent, Sarepta, Lexicon, Anacardio, and Akros; and has received grants from 3ive Labs, Abbott, Amgen, AstraZeneca, Bayer, Boehringer-Ingelheim, Bristol Myers Squibb, Cardurion, Cytokinetics, EBR Systems, Edwards, Medtronic, Novartis, V-WAVE, ViCardia, and Windtree Therapeutics. Drs Fiuzat have reported that she has no relationships relevant to the contents of this paper to disclose.
